# Co-Design and Evaluation Protocol for the RECOVER Model of Care After Childhood Cancer Treatment

**DOI:** 10.3390/healthcare13050454

**Published:** 2025-02-20

**Authors:** Natalie Bradford, Christine Cashion, Erin Sharwood, Shelley Rumble, Paula Condon, Danica Cossio, Helen Stratton, Stuart Ekberg, Remziye Semerci, Alison Bowers, Jason Pole, Kimberly Alexander

**Affiliations:** 1 Cancer and Palliative Care Outcomes Centre at Centre for Children’s Health Research, Queensland University of Technology, Brisbane, QLD 4001, Australia; ap.bowers@qut.edu.au (A.B.); k.alexander@qut.edu.au (K.A.); 2 Viertel Cancer Research Centre, Cancer Council Queensland, Brisbane, QLD 4215, Australia; 3 Children’s Health Queensland Hospital and Health Services, Brisbane, QLD 4101, Australia; christine.cashion@health.qld.gov.au (C.C.); erin.sharwood@health.qld.gov.au (E.S.); shelley.rumble@health.qld.gov.au (S.R.); paula.condon@health.qld.gov.au (P.C.); 4 Cancer Alliance Queensland, Metro South Hospital and Health Service, Woolloongabba, QLD 4102, Australia; danica.cossio@health.qld.gov.au; 5 School of Environment and Science, Griffith University, Brisbane, QLD 4222, Australia; h.stratton@griffith.edu.au; 6 Caring Futures Institute, College of Nursing and Health Sciences, Flinders University, Adelaide, SA 5042, Australia; stuart.ekberg@flinders.edu.au; 7 School of Nursing, Koc University, Istanbul 34450, Turkey; remziyesemerci@gmail.com; 8 Queensland Digital Health Centre, Centre for Health Services Research, The University of Queensland, St Lucia, QLD 4072, Australia; jason.pole@uq.edu.au

**Keywords:** model for care, survivor, childhood cancer, late effect, health services research

## Abstract

**Background:** Advances in diagnosis and treatment have significantly increased survival rates for childhood cancer, leading to a growing population of long-term survivors. However, these survivors face substantial physical and psychological sequelae that affect both the child and their family. We developed the RECOVER model of care to support childhood cancer survivors as they transition from the end of their planned treatment to survivorship, addressing the broader health and wellness needs beyond medical surveillance. The primary objectives are to assess the feasibility and acceptability of the RECOVER model of care in routine paediatric oncology practice. Secondary objectives include evaluating preliminary efficacy outcomes and identifying factors that influence the successful adoption and integration of the model. **Methods:** The study comprises a Type 2 Hybrid Implementation/Effectiveness non-randomised controlled trial to compare historical and prospective data. Quantitative data will assess feasibility, reach, effectiveness, adoption, maintenance, and implementation. The qualitative component will assess end-user acceptability and appropriateness through focus groups, surveys, and interviews. Quantitative and qualitative results will be integrated during the interpretation phase to provide complementary insights into the interconnected contextual factors that facilitate the model uptake. **Discussion:** The RECOVER model of care aims to offer a robust approach to survivorship care, facilitating the continuous monitoring and management of long-term and late effects in childhood cancer survivors. This model has the potential to significantly improve the quality of life and health outcomes for this vulnerable population by addressing their comprehensive needs in a timely and systematic manner.

## 1. Introduction

Advances in diagnosis and treatment have significantly increased survival rates for childhood cancer, with 86% of children in developed countries now surviving the disease [1]. However, the growing population of long-term survivors presents new challenges, as the cost of curing childhood cancer often includes substantial physical and psychological sequelae that affect both the child and their family [2].

Survivors of childhood cancer are at risk of serious late effects from their treatment, including the development of second cancers and chronic health conditions that can affect every organ and system in the body, ultimately diminishing quality of life [3,4,5,6]. Four in five childhood cancer survivors are at risk of these negative long-term sequelae, which can prevent a young person from reaching their full potential [7]. The healthcare needs of childhood cancer survivors have thus diversified and evolved, extending beyond medical surveillance and the narrow focus on survival to a more holistic approach aimed at improving the long-term health and quality of life of these individuals [3,4].

Our previous research highlighted significant challenges among this population in Australia. Young cancer survivors reported lower quality of life compared to population norms across physical, emotional, social, and functional domains. Many experience an average of nine persistent symptoms at moderate or higher intensity, even ten or more years after completing treatment [8]. Additionally, 36% of survivors were found to have overweight or obesity, suggesting poor lifestyle behaviours that may further contribute to chronic disease [9]. A substantial proportion also reported unmet needs, including the provision of timely information about long-term side effects (78%), understanding future outcomes (73%), managing side effects and symptoms (68%), and staying healthy (53%) [10]. Parents of children with cancer reported unmet needs at the end of treatment regarding information about what to expect, as well as high distress and complex feelings about managing their child’s ongoing healthcare [11,12].

The early detection and management of late effects are crucial in mitigating their impact, thereby significantly improving survivors’ overall health outcomes and quality of life [3]. However, existing models of survivorship care are often inadequate, often beginning five years post-treatment when patients are considered ‘cured’ and commonly discharged from oncology care. This model does not consider the needs of children or their families to transition to life following intensive periods of treatment, and care is not delivered in a consistent way with ad hoc referral practises. Consequently, an estimated 60–70% of survivors are lost to follow-up [7,13].

Substantial research evidence highlights the end of planned cancer treatment is a critical time point in the trajectory of care where care needs are not met [14,15,16]. Caregivers report heightened anxiety and fear as the end of treatment nears, with no clear plan beyond the intensive treatment phase [17]. There is a pressing need for more support, access to resources, and holistic survivorship care at the time of treatment completion. Delaying access to survivorship services represents a missed opportunity, as early intervention could minimise late effects and optimise long-term outcomes. A model of care at this critical juncture could reduce unmet needs and engage children and caregivers in proactive survivorship care [18].

Health services research is required to identify optimal models of care at this critical time at the end of planned treatment. Models of care need to facilitate empowerment and engagement, as well as care integration and coordination across specialist and primary care systems. This includes processes to improve screening with a structured assessment, tracking and reporting of health and information needs, and managing the transition between paediatric, adult, and primary care health services. This project explicitly addresses this gap by developing, implementing, and evaluating an integrated model of post-treatment care for children treated for childhood cancer.

The RECOVER model of care: This protocol paper describes the development of a new co-designed model of care, ‘RECOVER’, specifically tailored for childhood cancer survivors and the plans for evaluation. The broader study aims (to be reported at the completion of the study) are to

- Implement RECOVER into routine practice at a major tertiary children’s cancer centre.
- Assess the impact of the RECOVER model on outcomes, including caregiver information needs, experiences of coordinated care, and child and caregiver health-related quality of life.
- Evaluate the implementation costs, effectiveness, and outcomes of the RECOVER model of care from the perspectives of parents/carers and the health service.

## 2. Materials and Methods

Intervention development: The following sections provide an overview of the co-design of the RECOVER model of care, followed by the pilot outcomes and implementation effectiveness evaluation plans. The study protocol was registered with ClinicalTrials.gov (ID: NCT06586502).

Context and setting: The project is being undertaken at a major children’s tertiary hospital in Queensland, a geographically large and sparsely populated state in Australia. Queensland has centralised healthcare meaning many families must travel to access services. The Oncology/Haematology department receives approximately 260 new cancer-related referrals each year.

Patient and Public Involvement: Child cancer survivors and their carers (consumers) are involved at all stages of this research. They were represented on the original grant application for funding and participated in steering group meetings overseeing the conduct of this study. Consumers contributed to developing the RECOVER model of care through co-design, as outlined below, through participation in workshops, focus groups and interviews. During the trial, feedback will continue to be sought from consumers. Once the study is completed, lay summaries of the findings will be developed and disseminated to participants and relevant stakeholders.

Co-design of the RECOVER model of care: We used co-design principles [19] and implementation science methodologies to partner with childhood cancer survivors, caregiver consumers, and clinical experts. We sought to review current care gaps evident in the literature and practice and to develop a comprehensive model of care. The model was developed to incorporate the essential elements for comprehensive care outlined by the Australian Commission of Safety and Quality in healthcare, which include the following:

1. Clinical assessment;
2. Identification of goals of care;
3. Risk screening;
4. Comprehensive care plan development;
5. Care delivery;
6. Review.

Participants and setting: Participants included both healthcare providers and consumers. We aimed to use snowballing techniques to recruit approximately 20 stakeholders, including young cancer survivors, parents of young cancer patients and healthcare providers. Healthcare providers were selected based on their involvement in patient care and ability to provide valuable insights. They were recruited from the Queensland Children’s Hospital and included representation across medical, nursing and allied health specialists. Consumer participants were recruited through childhood cancer parent support groups and email contacts of the Queensland Collaborative for Cancer Survivorship. We exceeded our recruitment aim as outlined in Table 1.

**Figure 1:**
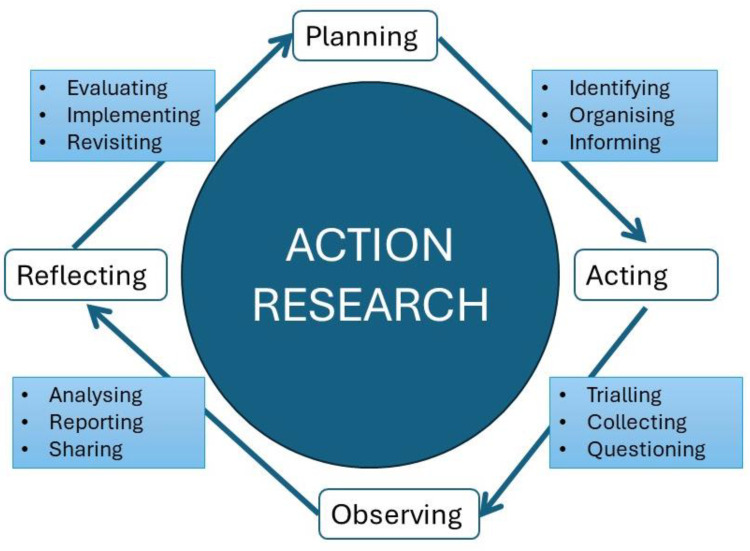
Action research diagram [20].

Procedures: The development of the RECOVER model of care followed a systematic and iterative approach guided by action research principles [20]. We conducted a series of workshops and focus groups (Figure 1) incorporating iterative cycles of planning, acting, observing and reflecting to identify and refine the processes required to deliver the essential elements of survivorship care. Discussions focused on the design of the model of care and the development of electronic resources to support the model. Stakeholders’ feedback was continuously reviewed, reflected upon, and incorporated to ensure the final model was acceptable and appropriate for consumers and healthcare providers [20].

**Table 1:** Participant characteristics.

**Healthcare Professionals *n* = 22**
Discipline
Nursing, *n* = 10; Includes oncology liaison nurses, neuro-surgical consultants, adolescent and young adult consultants, and regional case managers.
Allied Health, *n* = 8; Includes occupational therapy, pharmacy, speech pathology, psychology, neuropsychology, and ophthalmology.
Medical, *n* = 4; Includes paediatric oncologists, endocrinologists, and ophthalmologists, as well as general practitioners
**Parents of children with cancer *n* = 13**
Child’s Cancer type
Brain tumours *n* = 6
Solid tumours *n* = 7
Family location
Metropolitan *n* = 10
Regional *n* = 3
Child age at the time of study
5–9 years *n* = 3
10–18 years *n* = 10

Feedback and refinement: Feedback from healthcare professionals and carers of child patients was used to make continuous improvements to the process following the action research principles [20]. Feedback identified areas for improvement, and iterative refinements of processes were made. This ensured that the RECOVER model of care evolved based on real-world experiences and expert input. A summary of the issues identified and their implications for the RECOVER model of care are outlined in Table 2.

Building on these findings, the RECOVER model of care was finalised to address identified gaps and enhance the care of children at the end of planned treatment. The model is designed to integrate insights from the action research principles [20] and aligns with the Optimal Care Pathway for Paediatric Oncology [21], which provides a framework for delivering high-quality, evidence-based care. Patient/parent Report Outcome Measures (PROMs) are embedded to assess and monitor key outcomes. This alignment ensures that the model supports coordinated multidisciplinary care and reduces variations in practice, aligning with key service improvement priorities.

### 2.1. Description of Model of Care

**Figure 2:**
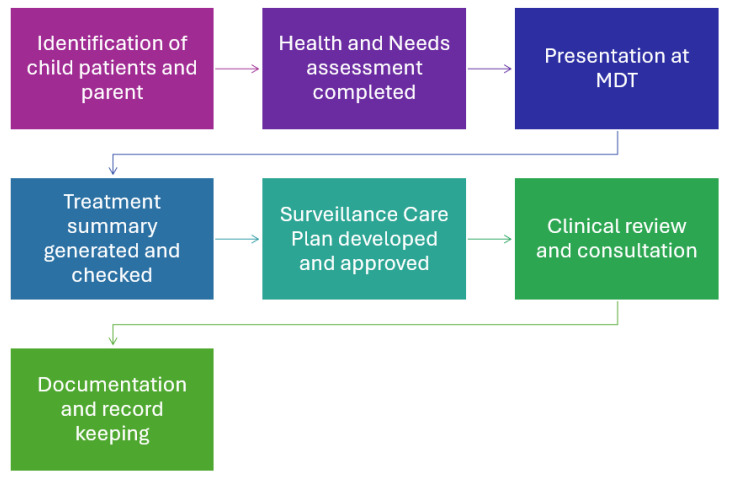
RECOVER end-of-treatment model of care.

The RECOVER model of care is intended to provide a structured, consistent approach to the management of children after acute cancer treatment, ensuring comprehensive follow-up and support throughout the transition from active treatment to survivorship. The model consists of seven key steps, each addressing critical aspects of survivorship care and facilitating communication and coordination among healthcare providers, patients, and families (Figure 2).

### 2.2. Model of Care Steps

1. Identification of Patients:
  The clinical team identifies patients approaching the ‘end of treatment’ phase optimally within two to six weeks prior to completion. This step ensures a timely transition to the surveillance phase.
2. Health and Needs Assessment:
  The RECOVER clinical nurse makes contact with the child patient and parent/carer to discuss the transition. A comprehensive ‘Health and Needs Assessment’ questionnaire is administered, evaluating unmet information needs, the child’s symptom profile (PROMIS paediatric proxy-item banks) [22] health status, and short-term goal setting using validated PROMs where appropriate (see the Supplementary Materials). This assessment can be conducted in person, via telephone, or via teleconference (Zoom or Microsoft Teams). The nurse also confirms the contact details of the family’s general practitioner (GP) or paediatrician for ongoing care and secures referrals as needed. If no GP is identified, the nurse facilitates a connection with a suitable GP. This step is crucial to understanding the needs of the child and family.
3. Multidisciplinary Team (MDT) Presentation:
  The patient’s case, including the outcomes from the Health and Needs Assessment, is presented at the regular MDT meeting after discussion of children on active treatment and palliative care. The MDT discusses ongoing surveillance plans, needs, symptom management intervention, and necessary referrals. Recommendations are made based on the patient’s health status, and tasks are allocated to appropriate healthcare professionals. This step ensures MDT input and that relevant health professionals are alerted to the child’s impending transition.
4. Treatment Summary Generated:
  The RECOVER clinical nurse prepares a detailed ‘treatment summary’ by collating data from administrative records, including QOOL, Electronic Medical Records (EMR), and the Chemotherapy Administration and Records Management (CHARM) (if applicable). Data include cumulative doses of chemotherapy, dates, field and dose of radiation and a summary of surgical procedures. The summary is checked for accuracy, reviewed and approved by the treating oncologist and ensures the child/family and relevant healthcare providers are provided with written information about the treatment received.
5. Surveillance Care Plan Development:
  The RECOVER clinical nurse develops a comprehensive Surveillance Care Plan for the patient and relevant healthcare professionals. This plan includes information gathered during the previous steps, a recommended timeframe for future examinations, reviews and investigations as recommended by the Children’s Oncology Group Long-Term Follow-Up Guidelines [23] and treating clinician practice. The care plan is written in accessible language. The Care Plan is approved by the treating oncologist. This step ensures a plan is in place with information summarising short-term goals, next appointments and follow-up plans, as well as relevant information about anticipated ‘late effects’ in the short term (<5 years).
6. Clinical Review and Consultation:
  A clinical review is scheduled with the patient’s caregivers at their nominated time, facilitated via telephone, telehealth or in person at the clinic. The RECOVER clinical nurse (and other relevant health professionals if needed) discusses the documents, addresses questions, resolves any barriers to care or adherence, and provides guidance. This step provides an opportunity to clarify the documents and information provided and ensure the family feels supported as they transition to surveillance.
7. Documentation and Record Keeping
  The Treatment Summary and Care Plan are then provided to the family in hard copy and electronic format and sent to relevant healthcare professionals (e.g., GP, Regional Hospitals) to ensure all parties have the necessary information. Electronic copies of the Treatment Summary and Surveillance Care Plan are uploaded into the child’s electronic medical records and the QOOL database for future reference to ensure continuity of care and ease of access.

### 2.3. User Testing of the RECOVER Model of Care

Procedures: User testing of the RECOVER model of care aimed to assess processes to integrate the model as a standard care practice, testing the processes in a real-world setting. Eligible patients who completed their treatment during this period were offered the RECOVER model of care. Research staff systematically observed and recorded activities during MDT meetings, reviewed both administrative and patient data, and entered these records into a REDCap study database, testing the study processes and procedures. Semi-structured interviews were completed with six parents to obtain their feedback, and timings for clinical activities were recorded. Minor modifications were made based on this feedback, for example, the timing of first approaching the family and the process for collating documentation for MDT meetings. Family feedback was generally positive.

*“I really liked the idea of having that like everything in that one document… handy, because throughout diagnosis and then different treatments and you’ve got little snippets of information here and there, and sometimes I sit and think, Oh! I need certain pieces of information and I have no idea of where to go looking for it. I am just happy to have all the information in one spot.”*

(ID2)

*“I thought it was helpful because, especially receiving the summary plan… where to go, like from there and like the doctors getting a copy of that plan so they know where we are at with [child’s] care and stuff. So, I think yeh it was pretty good. But I think it should have been sooner, um, yeh, that’s my personal opinion, like I think it should have been done sooner...like before we finished the final treatment kinda thing. Yeh, because it was at that stage that I was like, ok ...now what?”*

(ID4)

### 2.4. Implementation and Effectiveness Evaluation

A prospective consecutive group of child cancer patients and their parents will be recruited as the patients transition to the end of active treatment and surveillance to receive the RECOVER model of care. Data will be collected over 18 months to evaluate the immediate and longer-term impacts on carers’ information needs and experiences of integrated care. An embedded health services evaluation, informed by implementation science, will establish the feasibility, acceptability, impact, and value of the RECOVER model of care. This systems-level approach assesses processes, outcomes, and costs of interventions [24]. The study outcomes will be reported in peer-reviewed journals according to the Strengthening and Reporting of Observational Studies in Epidemiology Statement [25].

Study design: A Type 2 Hybrid Implementation/Effectiveness non-randomised controlled study design will be used to compare data from a historical control group (collected in a previous study, HREC/19/QCHQ/53816) with a prospectively recruited group of carers in the current study. Evaluation will focus on the implementation and preliminary efficacy of the model of care from the perspectives of patients/carers and the health service.

### 2.5. Procedures

Recruitment of parents of children with cancer: After active treatment, childhood cancer survivors will be stratified by clinical staff for risk of long-term and late effects according to validated protocols [26], identifying those who would benefit from early intervention and proactive management of the RECOVER model of care. While this process aims to identify those who would benefit most from the intervention, eligibility is broad, as outlined below, and children at lower risk are eligible if deemed clinically appropriate. As the model of care focuses on the transition from acute care services to community-based services, it is not suitable for children with uncertain prognosis, palliative care needs or other complexities. For these reasons, clinical staff review the suitability of all participants. Details of the parents of eligible children will be passed to the research nurse, who will provide written and verbal information and obtain informed consent to participate following standardised procedures [27]. We aim to recruit 40 parents.

Recruitment of healthcare professionals: Healthcare professionals (*n* ~ 10–15 medical, nursing, and allied health staff) involved in the care of children with cancer will be recruited through professional networks.

Study inclusion and exclusion criteria are presented in Table 3.

### 2.6. Data Collection

Parents: After providing consent, the participant will complete baseline measures. They will then receive the RECOVER model of care intervention as described above. Data will be collected to assess outcomes at three time points; Table 4 and Table 5. Semi-structured interviews will be completed with participants at follow-up to ascertain their feedback on acceptability and satisfaction with the model of care. Interviews will be completed by trained and experienced research staff and audio-recorded and transcribed verbatim.

Healthcare professionals: Interviews and/or surveys will be conducted with clinical staff to determine acceptability, satisfaction, barriers, and enablers for the model of care. Interview guides/surveys for healthcare professionals focus on implementation factors and are informed by domains from the PARiHS framework [28] to assess the local, organisational, and external contexts.

### 2.7. Measures and Outcomes

Implementation outcomes: Implementation evaluation is informed by Proctor’s implementation outcomes [29], and mapped to the RE-AIM [30] implementation framework (Table 3) and include the following:

- Feasibility and adoption assessed through (1) enrolment rates (proportion of eligible children’s parents who are (i) referred and (ii) consent to participate) defined a priori as greater than 50% of eligible parents enrolling in the model of care intervention [31]. (2) Proportion of enrolled participants provided with (i) Treatment Summary and (ii) Surveillance Care Plan. (3) Qualitative feedback mapped.
- Acceptability and appropriateness explored through (1) Rates of survey completion by parents at all times; considered acceptable if at least 70% of parents complete surveys [32]. (2) Parent/carer and health professional qualitative data collected through interviews. Parents will be asked about their satisfaction with the process, the relevance of collecting survey data, and the usefulness of the Treatment Summary and Surveillance Care Plan. Health professionals, including the family’s GP, will be asked about their satisfaction with the RECOVER model of care process and its relevance to clinical practice.
- Implementation Cost estimates will be based on the time the RECOVER Clinical nurse spends on clinical activities. The time spent will be prospectively recorded in a REDCap database during key activities, including review of medical records, development of Treatment Summary and Surveillance Care Plan, direct parent/child contact, preparation and attendance at MDT meetings, documentation and follow-up review. Monetary costs will be calculated using the 2024 wage rates for a Queensland Health Clinical Nurse with an additional 25% added to account for on-costs.
- Fidelity and Penetration will be assessed by an audit of the elements of the model of care completed for each child patient and fidelity to protocol as documented by the research nurse in the study database.

### 2.8. Effectiveness Outcome Measures

Preliminary effectiveness will be evaluated by measuring changes in several different outcomes, including care integration, information and service needs, parent/carer distress and child symptom burden, using validated measures described below. The schema for the timing of all measures is outlined in Table 4 and Table 5.

- Paediatric Integrated Care Survey (PICS): A family-reported survey tool of experiences of care integration [33]. The scale comprises five subdimensions: access to care; communication between care team members and parents/guardians; assessment and remediation of the impact of the child’s healthcare needs on the family; creation of short- and long-term care goals, and team functioning and quality of the care team [34]. We will compare outcomes from a historical control group (*n* = 100), matched for child age and diagnosis as closely as possible.
- Information and Service Needs: A bespoke survey was developed to assess the specific components of the RECOVER model of care from the perspectives of parents. The 15-item survey was developed from literature and includes domains for information needs; timing; utility and usefulness; and knowledge and confidence (see Supplementary Materials).
- Caregiver Quality of Life Cancer (CQOLC): This quality of life scale is a validated and reliable measure, comprising 35 items on a 5-point Likert scale, which we have widely used in other studies with this population [35]. We will compare outcomes of a historical control group (*n* = 100), matched for child age and diagnosis, as closely as possible.
- Distress Thermometer for Parents (DT-P): The DT-P is a valid and reliable short screening tool for identifying parental distress in the context of a chronically ill child (0–18 years). The DT-P consists of a thermometer score from 0 (no distress) to 10 (extreme distress) and a problem list (practical, social, emotional, physical, cognitive, and parenting domains) [36]. We will compare outcomes from a historical control group (*n* = 100), matched for child age and diagnosis as closely as possible
- Child Symptom Burden: Child symptoms are measured with the PROMIS paediatric proxy-item banks, which include domains for physical function, emotional distress, social peer relationships, fatigue and pain interference [22]. The measure is well validated and deemed clinically feasible and responsive in the paediatric cancer setting [37].

**Table 4:** Implementation outcome measures mapped to the RE-AIM framework.

Outcomes	Data Collected	Timing
RE-AIM DOMAIN: REACH		
% of referrals to the RECOVER model of care among eligible children	Research nurse completes in database	Audit at the completion of recruitment
Clinical and demographic characteristics of referred patients	Child medical records	At recruitment
Acceptability, satisfaction, barriers, enablers	Semi-structured interview/survey with parents and healthcare professionals	At study completion
RE-AIM DOMAIN: EFFECTIVENESS		
Changes in experiences of integrated care between the historical control group and RECOVER sample over time	Paediatric Integrated Care Survey (PICS) [33]	Baseline, 6 and 12 months
Changes in parent/carer distress and QoL compared to the historical control group and over time	Distress Thermometer–Parent [36]	Baseline, 6 and 12 months
Changes in child symptom burden over time	PROMIS paediatric proxy-item bank [22]	Baseline, 1, 6, and 12 months
Changes in information and service needs over time	The 15-item survey was developed for the study	Baseline, 6 and 12 months
RE-AIM DOMAIN: ADOPTION AND MAINTENANCE		
% eligible children/caregivers/provided with Treatment Summary and Care Plan	Research nurse completes in database	Audit at the completion of recruitment
% aware of the RECOVER model of care	Semi-structured interview/survey with healthcare professionals	At study completion
Fidelity to protocol—elements of model of care completed	Research nurse completes in database	Audit at the completion of data collection
Number of interventions/referrals/recommendations made per patient	Research nurse completes in database	Audit at the completion of data collection
RE-AIM DOMAIN: IMPLEMENTATION		
Acceptability, Appropriateness	Semi-structured interview/Survey with parents and healthcare providers	Parents at 3-month follow-up. Healthcare providers at the completion of data collection
% of patients presented at MDT meetings, the composition of MDT	QCH/QOOL records	Direct observation
% of Documentation of Treatment Summary and Care Plan in iEMR and My Health Record	QCH records—Research nurse completes in database	Audit completion of data collection
% of Treatment Summaries/Care Plans shared with GP	QCH records-Research the nurse completes in the database	At the completion of data collection
Implementation Costs	Research nurse records time for clinical activities in the database, including health and needs assessment, MDT, preparation of documents and communication with clinical staff	Audit at the completion of data collection

QCH: Queensland Children Hospital, QoL: quality of life, MDT: multidisciplinary team, QOOL: MDT software, iEMR: Integrated Electronic Medical Record, GP: general practitioner.

**Table 5:** Data collection schema.

Procedures	Pre-Enrolment	Baseline	1 Month	6 Months	12 Months
Eligibility Screening	X				
Recruitment	X				
Medical record review	X				X
Health and Needs Assessment		X			
Child Symptom Burden		X	X	X	X
Paediatric Integrated Care		X		X	X
Quality of Life—Parent		X		X	X
Distress Thermometer-Parent		X		X	X
Information and services needs			X	X	X
Exit interview/survey					X
In-depth interview (subset only)				X	X

### 2.9. Data Analysis

#### 2.9.1. Implementation Outcomes

We will use descriptive statistics for quantitative data for each of the constructs outlined and report these using means, proportions, standard deviations, or median and interquartile ranges, depending on the distribution of data. This will provide an objective measure of the implementation of the RECOVER model of care. Qualitative data from interviews or free-text comments will be analysed using thematic analysis supported by NVivo software v15 [38]. Patterns will be sought regarding perceived information and support needs, acceptability, satisfaction, information and educational needs, and barriers and enablers. Codes will be assigned to recurring themes. Consistencies and patterns in the themes will be identified and discussed with the research team and consumer advocates to achieve a consensus on findings. Findings from the formative work undertaken through these processes will be used to iteratively develop, design, and guide implementation strategies using expert recommendations for implementing change [39]. Fixed and variable costs of the model of care will be calculated to provide estimates of the resources required to sustain the model of care.

#### 2.9.2. Effectiveness Outcome Analysis

Quantitative analysis will involve collating and compiling data from various sources into the study database. Summary scores for measures at each time point will be calculated. Appropriate parametric or non-parametric tests will be used to compare the changes in outcomes between RECOVER participants and the historical control group from a previous study (*n* = 100) study, where participants will be matched as closely as possible for child age and cancer type. If possible, regression analysis will be completed to control for confounding variables.

Parent/carer knowledge, skills, and confidence will be assessed by analysing the survey responses with appropriate parametric or non-parametric tests depending on the data distribution. Data will be analysed using the IBM 28.0 SPSS programme.

#### 2.9.3. Ethical Considerations

Participation in all phases of this study is voluntary. Participants will be informed that participation will not affect their healthcare or professional relationships. If a participant is under 18, consent will be obtained from their parent/guardian. Written consent will be obtained at recruitment for all study phases. Participants will be informed they can withdraw from the study at any time, and their data will be deleted upon their request.

## 3. Discussion

Compelling data from large population-based studies highlight that childhood cancer survivors are a medically vulnerable population warranting survivorship services to optimise outcomes beyond cure [40]. However, translating the knowledge into clinical care is yet to be realised in many health services. Models of care after cancer treatment in childhood often focus on medical surveillance for cancer, neglecting the broader health and wellness needs of young survivors [3]. Moreover, many survivorship programmes do not commence until two to five years after cancer treatment, missing the opportunity to proactively address the needs of this medically vulnerable population [41]. The transition from acute treatment to survivorship care is often fragmented and fraught with difficulties as healthcare systems remain focused on the immediate needs of new cancer cases. This results in gaps in the provision of comprehensive post-treatment care. Parents of children are also calling for improvements; our earlier research identified that parents remain at an elevated risk for anxiety, depression, and symptoms associated with post-traumatic stress disorder [11]. Addressing information and support needs could mitigate this distress while also empowering parents with the knowledge they need to actively and confidently manage their child’s healthcare needs after treatment.

We aim to address these issues with the RECOVER model of care, which we will evaluate using a hybrid implementation effectiveness study. We have outlined the co-design, implementation and evaluation plans of RECOVER, which introduces a nurse-led programme of health and needs assessment, actions and resources to bridge the divide between cancer treatment and long-term survivorship. In doing so, we have addressed a significant deficit in the extant healthcare landscape for childhood cancer survivors [13].

### 3.1. Co-Design and Implementation

We described the process of co-designing the model with consumers and healthcare professionals using a human-centred approach and implementation science to maximise the potential success of the model in a real-world setting. The human-centred co-design approach embedded in the RECOVER model of care ensures that the needs of parents and children at the end of cancer treatment are central whilst simultaneously considering the constraints and resources available within the healthcare setting. The process of co-design and refinement of the model uncovered hidden assumptions, duplication of efforts, and unforeseen barriers. Identifying these was crucial to ensure that the final model could merge steps with existing processes and avoid creating unnecessary new steps.

### 3.2. Evaluation Plans

Our evaluation plans will assess implementation outcomes including the costs of sustaining the model beyond the life of the project. Using qualitative and quantitative research methods, we outlined our approach for assessing the impact and value of the project. Anticipated outcomes include the establishment of a comprehensive model for end-of-treatment care to effectively address the diverse needs of parents and children after cancer treatment.

### 3.3. Implications for Clinical Practice

The RECOVER model of care has considerable implications for clinical practice. Nurses and other healthcare professionals play a crucial role in this model, from conducting health assessments to coordinating care plans. The model emphasises the importance of continuous monitoring and management of treatment-related effects, which can lead to improved health outcomes and enhanced quality of life for survivors. Integrating this model into clinical practice can also improve communication and coordination among healthcare providers, patients, and families, fostering re-engagement with primary care providers and leading to a more patient-centred approach to care. Implementing these alternative models on a broader scale will require adequate support from non-oncologist care providers and endorsement by cancer teams and their patients [42].

### 3.4. Strengths and Limitations of This Study

One of the strengths of the RECOVER model of care is its comprehensive, multi-phase approach integrating feedback from a diverse group of stakeholders. The involvement of both consumers (parents and families) and healthcare professionals in co-design ensures the model is tailored to the real-world needs and constraints of its users. Further, by implementing science principles, this study maximises the potential for success in real-world application and sustainability.

Our study also has limitations. First, its potential bias is introduced by the voluntary participation of both parents and health professionals whose decision to participate may be influenced by their interest or personal commitment to the project, possibly affecting the generalisability of the findings. Additionally, this study relies heavily on self-reported data from surveys and interviews, which may be subject to recall bias or social desirability bias, potentially impacting the accuracy of the reported outcomes. Finally, this study’s implementation and evaluation phases are context-specific to Queensland, Australia, which may limit the applicability of the findings to other regions or healthcare systems with different resources, cultural contexts, or healthcare practises.

## 4. Conclusions

As the population of childhood cancer survivors continues to grow, it is imperative healthcare systems evolve to meet their ongoing needs. This protocol outlines the co-design of a model of care for children at the end of their cancer treatment, incorporating actions of patient/parent-reported outcomes. The objective is to integrate health and information needs within a collaborative service paradigm to assess and respond to these needs comprehensively and systematically. The model has the potential to re-establish relationships with primary care and facilitate the early detection, referral, and management of treatment-related effects among childhood cancer survivors. The model of care will be tested in a hybrid implementation effectiveness study, assessing feasibility, acceptability, effectiveness, and costs.

## Figures and Tables

**Table 2:** Issues and Implications for the RECOVER model of care.

Issue	Implication
Multi-disciplinary Team (MDT) involvement and communication needs	
The need for seamless collaboration was identified. Doubts about the feasibility of involving all team members and how this might impact the quality of care. “How can this actually happen; what is feasible in the real world?”	Concern about practical implementation, with some suggestions that a virtual MDT could work if organised properly. Although coordination between team members is needed, logistical challenges may hinder this
Communication challenges between healthcare providers and families	
Recurring concerns were voiced regarding the effectiveness of communication between healthcare providers, particularly regarding updating families and GPs on treatment and care plans. This is crucial in rural and regional settings.	Highlights the difficulties of relaying information effectively and the need for more structured, reliable methods of communication.
The role of allied health in survivorship care	
Several clinicians highlighted the underutilisation of allied health, such as pharmacists in the care team. There is a strong call for their greater involvement, especially in managing long-term side effects to improve survivorship care.	The expertise and knowledge of allied health staff are an underused resource that could fill a critical gap in care, particularly for managing long-term issues.
Resource limitations and gaps in support services	
Need for resources and staffing, especially in areas such as pharmacy, psychosocial support, and survivorship planning. Discussions about gaps in staffing and service availability negatively impact families. “Families would really benefit from a check-in at 12 and 24 months—life doesn’t always get easier.”	Although there is enthusiasm and willingness to support families, the lack of funding and resources creates substantial barriers to offering holistic care.
Family needs and psychosocial support	
Families’ psychosocial needs, including support for siblings and mental health, are crucial but often unmet. Families express a need for ongoing psychosocial support, particularly as the child reintegrates into normal life.	These discussions highlighted that while clinical treatment is paramount, emotional and psychological support for families should not be overlooked.
Survivorship care planning	
Ongoing challenges in how survivorship is managed, both in terms of the terminology used and the timing of the provision of care plans and follow-up services. Recognition there is a ‘grey area’ following the end of treatment. Families have voiced it would have been helpful to have had the connection to services without a large gap in continuity of care.	This reflects the uncertainty families experience in transitioning from active treatment to survivorship care, emphasising the need for a more structured, continuous approach to post-treatment planning.

**Table 3:** Inclusion and exclusion criteria of the study.

Participants	Inclusion Criteria	Exclusion Criteria
Parents/carers of children treated for cancer	Child Any diagnosis of cancer; Aged 0–18 years at the time of diagnosis; Beginning surveillance or nearing the end of planned treatment or within two years of completion of treatment. Parent/carer Over 18 years; Able to read and understand English; Willing to freely provide informed consent and participate in study procedures;	Child/parents/carers deemed not suitable by clinical teams. Child with uncertain prognosis or palliative care needs.
Health professionals	Clinicians (doctors, nurses, and allied health professionals) who provide care through Queensland Health public hospitals; The participants’ nominated family general practitioner.	None; all clinicians who meet the inclusion criteria are eligible to participate.

## Data Availability

The datasets presented in this article are not readily available because the data are part of an ongoing study. Final data will be available upon request with appropriate approvals.

## Supplementary Materials

The following supporting information can be downloaded at: https://www.mdpi.com/article/10.3390/healthcare13050454/s1, Timeline of Key Milestones; Health and Needs Assessment; Symptom Survey.
