# Supplementary material for: Co-Design and Evaluation Protocol for the RECOVER Model of Care After Childhood Cancer Treatment
Source: Healthcare (Basel). 2025 Feb 20;13(5):454. doi: 10.3390/healthcare13050454 (PMC11899481; doi:10.3390/healthcare13050454)
Supplement: Supplementary file 1 [file healthcare-13-00454-s001.zip › healthcare-3451407-supplementary.pdf]

Supplementary Files:

Timeline of Key Milestones

Key milestones and decision points were tracked over the course of 18 months (July 2022 – Dec 2023). Below, we outline the chronological progression of activities and critical developments during co-design:

Initial Stakeholder Engagement and Feedback (July – October 2022):

- July 2022: Initial presentations were made at Oncology clinical research meetings, introducing the concept of the RECOVER project to stakeholders, including consumer representatives and clinicians. Early consumer feedback was collected, identifying initial concerns regarding current care pathways and resource gaps.

- August 2022: Feedback from stakeholders highlighted potential issues with the proposed direction of the model, prompting adjustments in the project scope.

- September 2022: Following concerns raised during the feedback sessions, a significant decision was made to abandon the original plans to develop an electronic portal due to sustainability and logistical issues, redirecting focus towards a more user-centred approach.

- October 2022: Emphasis shifted towards further refining care pathways based on ongoing stakeholder input, setting the stage for collaborative design efforts.

Development and Refinement of the Model (November 2022 – May 2023):

- November 2023: Iterative development began on the QOOL Multi-Disciplinary Team (MDT) platform [1] and initial contributions were made towards developing a survivorship screen for clinical use and survivorship care plans. Plans were to automate the calculation of cumulative chemotherapy. Early versions of resources were trialled during stakeholder workshops.

- February 2023: Draft treatment summaries and care plans were developed from existing resources.

- May 2023: Feedback sessions focused on resource modifications, addressing concerns of duplication and enhancing usability for families and healthcare providers.

Model Optimisation and Resource Integration (June – December 2023):

- June 2023: Development efforts concentrated on integrating resources to electronic versions and exploring opportunities for process automation. Chart audits were conducted to assess variations in referral pathways and timing of care delivery.

- July 2023: Pilot testing of the automation of radiotherapy and cumulative chemotherapy dose for treatment summaries revealed data inaccuracies. This prompted a review of data entry processes and an assessment of the feasibility of accurately automating treatment summaries.

- September 2023: Further challenges related to the limited resources available within the project to fully automate accurate Treatment Summaries and Care Plans using QOOL

were identified. Alternative proposals of a dashboard to support real-time tracking of patient care pathways were proposed.

- December 2023: Documentation to support the Model of Care was finalised to include a comprehensive health and needs assessment interview, Treatment Summaries and Care Plans.

Finalisation and Stakeholder Validation (January – June 2024):

- January 2024: The final draft of the resources were shared with stakeholders for review. A dashboard visualisation was developed in Excel alongside a survivorship screen in the QOOL database.

- February 2024: Stakeholder engagement continued with a focus on refining referral processes for patients with diverse conditions, enhancing the inclusivity of the care model.

- March 2024: Interviews with parents, addressing previous feedback and confirming the acceptability of the Model of Care completed. Ongoing challenges with data collation were discussed, and iterative feedback was used to adjust the Model of Care, e.g. timing for presentation to the multidisciplinary team.

RECOVER HEALTH & NEEDS ASSESSMENT

Date:	
Child's name:	
Parent/Carers present:	

General Distress – Distress thermometer (Carer)

On a scale of 1-10 how much distress has the caregiver felt over the past week?<br><br>(write number from 0 to 10)	Distress level:<br><br>Comments: 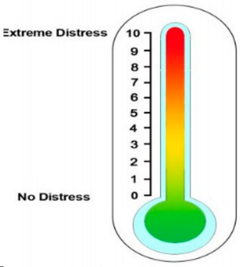

Community healthcare team contact details

Current GP contact details:	
Regional Centre/Paediatrician contact details (if relevant):	
Community therapist contact details: (if relevant)	
Community counselling/psychology contact details: (if relevant)	

Specific areas of Concern or Adjustment

(tick all of concern and briefly describe issue)

**Physical**
☐Fatigue, sleep problems
☐ Side effects from treatment
☐ Headaches
☐ Pain
☐Dizziness
☐Function issues/impairment
☐Balance, coordination.
☐Seizures
☐Eyesight
☐ Hearing
☐ Speech,
☐ Dental concerns
☐Endocrine/Metabolic concerns,
☐ Weight loss/gain
☐ Growth & development
☐Peripheral neuropathy

Cognitive /Learning
☐Memory issues
☐ Taking longer to complete activities
☐ Short attention span
☐Returning to school, catching up on class work,
☐ Concerns regarding peer acceptance,
☐ Communication with school
☐Learning support required
☐ RMD Learning support program
☐ Brainchild Learning support
☐Behaviour changes

Emotional, personality and behavioural changes
☐Anxiety
☐ Sadness
☐ Anger
☐Feeling frustrated
☐ Feeling helpless

Social, Hobbies & Peer Relationships
☐Hobbies
☐ Peer networks
☐Feeling isolated from friends
☐Feeling different
☐Getting teased, bullying

Psychology
☐Seen a psychologist
☐Follow up planned
☐Concerns with procedural related anxiety
☐Concerns with thoughts/feelings/ related to diagnosis and treatment

Lifestyle concerns
☐Healthy living
☐ Diet
☐ Weight management
☐ Exercise
☐ Injury avoidance
☐ Sun protection
☐ Me time
☐Risk taking
☐ Not attending appointments

Impact of experience
☐Change in identity
☐ Medical trauma
☐Change in body image

Fertility, sexuality & intimacy
☐Fertility concerns
☐ Fertility preservation options accessed
☐ Concern with discussing fertility status with others
☐ Gender identity
☐ Intimacy concerns

Family
☐Worry about impact on family
☐ Lack of support from extended family/friends,
☐ Lack of understanding from extended family/ friends
☐Financial strains
☐Challenge to families' faith, spirituality or strengthening of
☐Identifying needs of other family members

After Treatment Concerns
☐Reluctance to share diagnosis
☐Concerns about PTSD
☐Worry about long term effects
☐Worry about return of tumour/cancer
☐Survivor guilt

Medications
☐Ensure family aware of current medications
☐ Consider benefit of pharmacy plan
☐Immunisation plan in place

Other concerns:

Carer's confidence to manage any health issues/complications

Goals for the future: *(priorities/goals of the child/family)*

Contact numbers /websites requested:

Further information requested:

Consent for contact details (phone number & email) to be placed on the Treatment Summary?

(complete consent form if ‘yes’)

☐Yes

☐No
